# Health-related quality of life in children and adolescents living with Gaucher disease and their parents

**DOI:** 10.1080/21642850.2018.1462705

**Published:** 2018-04-12

**Authors:** Eduardo Remor, Antonio Baldellou

**Affiliations:** a Faculty of Psychology, Universidad Autónoma de Madrid, Madrid, Spain; b Institute of Psychology, Universidade Federal do Rio Grande do Sul, Porto Alegre, Brazil; c Unidad de Enfermedades Metabólicas, Hospital Universitario “Miguel Servet”, Zaragoza, Spain

**Keywords:** Health-related quality of life, assessment, genetic disorders, Gaucher disease

## Abstract

**Objective:** The health-related quality of life (HRQoL), clinical status and perceived burden of disease in children and adolescents with Gaucher Disease (GD) were assessed.

**Method:** A Spanish multicenter collaboration study involving 13 hospitals was performed to evaluate pediatric patients with GD (*n* = 17, ages 5–18; *n* = 3, ages 2–4) and their parents (*n* = 20) using a HRQoL measure (PedsQL 4.0) and a survey on the perceived burden of the disease. Three children under five years old were evaluated by parent proxy-report. Relevant medical and socio-demographical characteristics were recorded.

**Results:** Sixty-nine percent of the participants with GD had mild and 31% had moderate severity level, all receiving enzyme replacement therapy (ERT). HRQoL was associated with the severity score index and was adjusted for age. Age was related to school functioning (older patients had lower scores), and female patients reported worse school functioning than males. Symptoms such as bone, joint or abdominal pain, bleeding, and fatigue were negatively associated with HRQoL. Perceptions of the burden related to GD, such as feeling ill and feeling sad, were negatively associated with HRQoL. Although the PedsQL scores of children and parents showed concordance, patterns of association between symptoms and perceived burden differed between children and parents. No associations were observed between HRQoL scores and time on ERT or ERT dosage.

**Conclusion:** HRQoL perceptions were affected by clinical status, observable and subjective symptoms, feelings of burden related to the disease, and patient characteristics (e.g. age and gender). Aspects of the disease that affect HRQoL may be perceived differently by children and parents.

## Introduction

Gaucher Disease (GD) is rare, genetic metabolic disorder that affects 1 in 149,000 individuals in the Iberian Peninsula, similar to that of other European populations (Giraldo et al., 2016). GD is the most common lysosomal storage disorder and results from a specific enzyme deficiency in the body that is caused by a genetic mutation in the gene encoding the lysosomal enzyme acid beta glucosidase (Deroma et al., 2013). This disease is classified into 3 clinically recognized types based on the presence and degree of nervous system involvement. GD Type 1 is the most common and is characterized by a lack of central nervous system involvement, whereas GD types 2 and 3 are characterized by acute and chronic neurologic symptoms, respectively (Baldellou, Dalmau, & Sanjurjo, 2016; Giraldo et al., 2016). The course of GD is variable, ranging from no outward symptoms to skeletal problems, liver or spleen damage, bleeding, severe disability and death (Erikson, Karlberg, Skogman, & Dreborg, 1987; Goker-Alpan et al., 2008). The most common symptoms include enlargement of the liver and spleen, anemia, reduced platelets (resulting in easy bruising and long clotting times), bone pain, bone infarctions that often lead to damage of the shoulder or hip joints, and generalized bone demineralization (osteoporosis). The weakening of the bones can subsequently lead to spontaneous fractures (Milligan, Hughes, Goodwin, Richfield, & Mehta, 2006).

Testing is available to identify potential parents who are carriers of the gene and to accurately diagnose people who have the disease. An effective enzyme replacement therapy (ERT) has been available since 1991 (Baldellou et al., 2004; Grabowski et al., 2004; Masek et al., 1999; Milligan et al., 2006). Although the treatment is highly efficacious, it is also intrusive and expensive and requires patients (and families) to restructure their daily lives (Packman, Wilson Crosbie, Riesner, Fairley, & Packman, 2006).

Since the National Institutes of Health (NIH) Consensus Panel on Gaucher Disease (McCabe, Fine, & Golbus, 1996) and the subsequent publication of experts’ consensus statements (Baldellou et al., 2004; Grabowski et al., 2004), the importance of assessing health-related quality of life (HRQoL), physical functioning, disease burden, satisfaction with treatment, and impact on family functioning was recognized. Collecting these indicators may help health care professionals define the appropriate clinical indications for treatment and establish uniform methods to optimize outcome assessments. Additionally, offering more personalized interventions based on outcomes and incorporating a holistic approach in patient care are of paramount importance.

Studies investigating the HRQoL with GD have been mostly habitual and included primarily adult patients (e.g. Damiano, Pastores, & Ware, 1998; Hayes, Grinzaid, Duffey, & Elsas, 1998; Masek et al., 1999; Oliveira et al., 2013). Past studies found that domains of life, such as physical functioning-related daily activities at school, home or work, were affected by the disease. Social life and activities with family and friends were diminished by a lack of energy. Emotional health was damaged by low self-esteem, anger, and symptoms of anxiety or depression related to awareness of the limitations imposed by the disease. Stress associated with treatment burden, time consumption and cost were also reported. Patients perceived that the disease limited their options in life and impacted their future plans (Hayes et al., 1998).

Comparing patients with GD to the general population revealed that the HRQoL domains were significantly lower in patients (i.e. lower physical functioning, the presence of physical limitations, higher pain, lower general state of health, less vitality, and lower social functioning) (Damiano et al., 1998). Patients who had access to ERT perceived significant improvements in HRQoL after treatment (i.e. increased in functional capacity and vitality, perceived physical and mental health, and improved social functioning) (Masek et al., 1999). The results relating to the benefits in HRQoL related to ERT have been replicated (Deroma et al., 2013; Giraldo, Pocoví, Pérez-Calvo, Rubio-Félix, & Giralt, 2000; Weinreb et al., 2007). Furthermore, treated patients may present HRQoL scores similar to those of the general population, as demonstrated in previous past studies (Pastores, Barnett, Bathan, & Kolodny, 2003). However, despite treatment, patients with GD report concerns beyond quality of life, such as difficulties coping with the diagnosis, the impact of pain on daily life and recreational activities, mood changes and anxiety, and a negative outlook on life (Packman, Crosbie, Behnken, Eudy, & Packman, 2010). Psychological complications, such as somatic concerns, sadness, emotional distress, tension, and increased physical symptoms and suffering in stressful situations, have also been described (Packman et al., 2006).

To the best of our knowledge, no study has addressed the description of HRQoL in pediatric populations with GD. Indeed, research has been more focused on neuropsychological elements (Capablo et al., 2008), intelligence (Erikson et al., 1987) and cognitive functioning (Goker-Alpan et al., 2008) in children and adolescents with GD. However, according to the literature (Grabowski et al., 2004), children with GD may experience considerable psychosocial problems, which may impair their quality of life. As with any debilitating disease, they may have difficulty adjusting to the disease and may experience feelings of distress, including anger, denial, fear, insecurity and isolation. Older children and adolescents may also develop behavioral problems. Organomegaly, growth abnormalities and delayed puberty may affect body image and social functioning and lead to self-stigmatization, decreased self-esteem and psychosexual difficulties in older children. An increasing awareness of ‘being different’ may be amplified when fatigue and/or restrictions on physical activities due to splenomegaly and/or bone disease prevent children from participating in sports and other related activities. Furthermore, chronic pain and fatigue may affect school performance (Grabowski et al., 2004).

Nevertheless, according to a previous systematic review (Cohen & Biesecker, 2010) and to the best of our knowledge, standardized generic measures of HRQoL have not been applied to pediatric patients with GD. The goal of the current study is to describe the HRQoL and perceived burden of disease of pediatric patients and their parents. Due to exploratory characteristic of the study no formal hypotheses were formulated.

## Methods

### Participants

The participants were pediatric patients aged 3–18 years with physician-diagnosed GD and their parents. The average age of the children was 10.3 (SD 4.6). Eleven patients were male, and nine were female. The socioeconomic statuses of the families were as follows: 10% low income, 40% medium-low income and 50% medium-high income. The severity of the disease was mild in 69% of the participants and moderate in 31% of the participants. All patients presented with the non-neurological type (i.e. the type that lacks central nervous system involvement). The majority of patients received treatment every 2 weeks. The time on ERT ranged from 2 to 115 months (M 51.9; SD 38.4). Detailed patient characteristics are presented in Table 1.

**Table 1.** Sample characteristics.

Characteristics	Mean (SD), Range	Median (IQR)	(*n*) %
Patient age (years)	10.3 (4.60), 3–18	10 (7.88)	
Age groups			
2–4			3 (15)
5–7			5 (25)
8–12			5 (25)
13–18			7 (35)
Gender			
Male			11 (55)
Female			9 (45)
Severity level (GD)			
Mild			11 (69)
Moderate			9 (31)
Severe			0 (0)
SES (family self-report)			
Low			2 (10)
Middle-low			8 (40)
Middle-high			10 (50)
High			0 (0)
Treatment frequency (past 4 weeks) (*n* = 18)	1.94 (0.24), 1–2	2 (0)	
Time on ERT (months) (*n* = 18)	51.94 (38.39), 2–115	37.5 (62)	
Current treatment dose (Un/kg) (*n* = 18)	784.06 (949.59), 30–3000	90 (1469)	
Age-adjusted SSI (*n* = 18)	7.17 (5.59), 3–29	6 (1.75)	
Splenectomy			
Yes			1 (5)
No			17 (85)
Missing values			2 (10)
Patients by region of Spain			
Andalucía			2 (10)
Aragón			5 (25)
Castilla y León			1 (5)
Extremadura			4 (20)
Rioja			1 (5)
Madrid			3 (15)
Murcia			1 (5)
Valencia			3 (15)

IQR: interquartile range; ERT: enzyme replacement therapy.

### Measures

#### Sociodemographic and clinical-related variables of the patients

Age, gender, socioeconomic status (SES), GD severity, treatment frequency, time on ERT, and treatment dose were recorded from medical charts.

#### PedsQL 4.0 generic core scales

The 23-item PedsQL 4.0 Generic Core Scales (Varni, Seid, & Kurtin, 2001; Varni, Seid, & Rode, 1999) constitute a multidimensional instrument that encompasses four domains: Physical Functioning (8 items), Emotional Functioning (5 items), Social Functioning (5 items), and School Functioning (5 items). The Physical Health Summary Score is the same as the Physical Functioning Scale. To create the Psychosocial Health Summary Score, the mean is computed as the sum of the items divided by the number of items answered in the Emotional, Social, and School Functioning Scales. The scales use analogous child self-report and parent proxy report formats for children aged 5–18 years and a parent proxy report format for children aged 2–4 years. The questions assess how much of a problem each item has been during the previous month. Items are reverse-scored and linearly transformed to a scale of 0–100, with higher scores indicating better HRQoL. Scale scores are computed as the sum of the items divided by the number of items answered (which accounts for missing data). If more than 50% of the items in the scale are missing, then the scale score is not computed (Varni et al., 2001, 2015). The global reliability (23 items) of the current sample was 0.87 for the child self-report form and 0.96 for the parent proxy report form (see reliability details in Table 2).

**Table 2.** Descriptive scores and reliability of the PedsQL 4.0 applied to pediatric patients with GD.

PedsQL 4.0	Number of items	Reliability α	*N*	Min.	Max.	Mean	SD	Median	IQR
*Child self-report*									
Summary Score | Total Score	23	.87	17	61.96	97.83	86.76	10.89	91.30	14.67
Physical Health Summary Score	8	.77	17	59.38	100	90.25	12.44	93.75	14.06
Psychosocial Health Summary Score	15	.79	17	58.33	96.67	84.90	11.20	88.33	13.33
Emotional Functioning	5	.58	17	45	100	86.18	15.26	90	20
Social Functioning	5	.57	17	50	100	87.94	13.81	90	17.50
School Functioning	5	.54	17	55	95	80.59	10.73	80	12.50
*Parent proxy-report*									
Summary Score | Total Score	23	.96	20	40.22	100	84.30	17.22	91.84	28.32
Physical Health Summary Score	8	.93	20	31.25	100	86.72	19.89	95.31	25.78
Psychosocial Health Summary Score	15	.94	20	41.67	100	83.01	17.40	91.67	25.77
Emotional Functioning	5	.74	20	45	100	83.50	16.94	87.50	28.75
Social Functioning	5	.88	20	40	100	87.50	19.30	100	20
School Functioning	5	.81	20	30	100	77.50	22.14	85	13.75

α: Cronbach’s alpha; SD: standard deviation; IQR: interquartile range; PedsQL (Scores range from 0 to 100; high scores indicate better health-related quality of life).

#### Age-adjusted severity score index (SSI)

The age-adjusted SSI of Zimran et al. (1989) was assessed and computed by a pediatrician and used to evaluate the extent of organ involvement, symptoms, clinical status and phenotypes. Higher scores indicate worse clinical status and prognosis.

#### Perceptions of the burden of the disease and treatment survey

A 10-questions survey developed ad hoc by the authors (with the review of the Spanish Group of Pediatric Experts on Gaucher Disease) was completed by patients and their parents to assess their perceptions of the burden of the disease. The survey included questions about perceptions of symptoms related to the illness (e.g. bone and/or joint pain, abdominal pain, bleeding, fatigue), perceptions of the burden of the disease (e.g. feeling ill and feeling sad), and beliefs and expectations about the treatment (e.g. the level of information about the treatment, the level of confidence in the doctors, treatment as protection against illness progression, and feelings of anxiety during treatment infusion at the hospital). Answers were computed in terms of presence/yes or absence/no.

### Procedures

This work was developed to assess the HRQoL and perceived burden of the disease in children living with GD (and their parents) in Spain. As at the time the study started, studies using questionnaires with no invasive measures were not being evaluated by the ethics committee at our university, the proposal was submitted and approved, regarding quality and ethics, by the Spanish Group of Pediatric Experts on Gaucher Disease. Participants were recruited between 2004 and 2006. A group of medical professionals (pediatricians) following children and adolescent patients with GD from 13 hospitals in Spain were contacted to participate in the study. Patients who met the eligibility criteria were identified by the pediatrician during a regularly scheduled hospital visit. Eligible parents and patients were approached by a medical resident or nurse and given a detailed description of the study. All invited participants agreed to participate. The caregiver who attended the clinic visit was asked to complete the study protocol. If multiple caregivers were present, they were asked to choose one primary caregiver to complete all of the measures. Questionnaires were self-administered for parents and children/adolescents aged 8–18 years, and for children aged 5–8 years the questions from the research protocol was read by the interviewer. The PedsQL reports for children aged 2–4 were only answered by parents. Medical records (i.e. SSIs and clinical-related variables) were provided by the pediatrician (however, some data were missing for two patients). Children and adolescents (5–18 y.o.) gave verbal assent after procedures were explained, and one parent or legal guardian provided written informed consent. For children 2–4 years old, only parents provided written informed consent.

### Data analysis

The data were examined for missing information, outliers, and normality. Because the variables did not follow a normal distribution, they were expressed in terms of the mean (SD), range, median and interquartile range (IQR). Discrete variables were expressed as frequencies and percentages. Considering the small sample size and the fact that the variables did not show a normal distribution, all statistics were calculated using nonparametric tests (i.e. Spearman, Mann–Whitney, and Wilcoxon). The effect size of the Mann–Whitney *U*-statistic was calculated using an equation to convert a *z* score into a de effect size estimate, *r*, according to Rosenthal (1991, p. 19). Cronbach’s alpha was used to determine the scale internal consistency reliability. The statistical analyses were conducted using SPSS 21.0 version software (SPSS, Inc., Chicago, IL, USA).

### Ethics statement

All aspects of the project and study were conducted in compliance with The Code of Ethics of the World Medical Association (Declaration of Helsinki). The participants completed the study protocols in the hospital and did not receive any payment for their participation.

## Results

### HRQoL in children living with GD as measured by PedsQL 4.0

HRQoL values, as measured by PedsQL 4.0 descriptive scores (i.e. mean, SD, min.-max., median, and IQR) for the current sample are presented in Table 2.

### Children’s and parents’ HRQoL perspectives

To assess the similarities or differences in their HRQoL perspectives, scores reported by children and their parents were compared using the non-parametric two-related samples Wilcoxon signed-rank test. Although children reported slightly lower scores in their perceptions of HRQoL (according to Physical Health and Psychosocial Health summary scores) relative to their parents’ assessments, the differences were not statistically significant across all matched domains (All *ps* > 0.05, see supplementary material).

### Characteristics of the patients and treatment relative to perceived HRQoL

Age was only related to school functioning (child self-report, PedsQL). Older children reported worse school functioning (rho = −0.52, *p* = 0.047), and female patients reported worse school functioning than male patients (*U* = 15.50, *p* = 0.044, *r* = −.49). No associations were found in terms of the disease severity (mild/moderate), SES, treatment frequency, time on ERT, or current treatment dosage with the HRQoL scores measured based on child self-reports and parent proxy reports on the PedsQL 4.0.

### Association between GD clinical indicators and HRQoL

Table 3 shows the associations between the age-adjusted disease severity and the HRQoL measured based on PedsQL 4.0 scores. The severity score index (SSI; higher scores indicate worse clinical status and prognosis) was related to all domains of the PedsQL, except for emotional and school functioning scores according to the children’s perspectives and school functioning from the parents’ perspectives.

**Table 3.** Correlation between a GD clinical indicator (SSI) and HRQoL scores.

PedsQL 4.0 Subscales	Severity Score Index age-adjusted
	rho (*p*)
Child Self-Report (*n* = 15)	
Summary Score | Total Scale Score	−.79 (.001)
Physical Health Summary Score	−.63 (.011)
Psychosocial Health Summary Score	−.68 (.005)
Emotional functioning	−.30 (.275)
Social functioning	−.79 (.000)
School functioning	−.50 (.057)
Parent Proxy-Report (*n* = 18)	
Summary Score | Total Scale Score	−.52 (.010)
Physical Health Summary Score	−.49 (.037)
Psychosocial Health Summary Score	−.63 (.005)
Emotional Functioning	−.69 (.002)
Social Functioning	−.62 (.006)
School Functioning	−.37 (.129)

Rho: Spearman’s rank correlation; SSI (higher scores indicate worse clinical status and prognosis); PedsQL (high scores indicate better health-related quality of life).

### Association of symptoms and perceived burden related to GD with HRQoL

Table 4 shows the statistically significant associations (and effect size) of symptoms (i.e. bone and/or joint pain, abdominal pain, bleeding, and fatigue) and perceived burden related to Gaucher (i.e. feeling ill and feeling sad) with HRQoL scores, as measured by the PedsQL.

**Table 4.** Associations of symptoms and perceived burden related to GD with HRQoL (PedsQL) scores.

PedsQL 4.0 Subscales	Symptoms and perceived burden associated with the illness					
	Mdn (yes/no), Mann-Whitney *U* test (*p*), *r*					
	Bone and/or joint pain	Abdominal pain	Frequent or abnormal bleeding (any type)	Fatigue	Feeling ill (I could not do what I wanted)	Feeling sad
*Child self-report* (*n* = 17)	yes = 9, no = 8	yes = 8, no = 9			yes = 6, no = 11	yes = 3, no = 14
Summary Score | Total Scale Score	ns	ns	ns	ns	79 / 92 *U* = 7.00 (.008), *r* = −.64	74 / 91 *U* = 2.00 (.016), *r* = −.58
Physical Health Summary Score	ns	ns	ns	ns	Ns	62 / 94 *U* = 1.00 (.009), *r* = −.63
Psychosocial Health Summary Score	87 / 92 *U* = 14.00 (.033), *r* = −.51	ns	ns	ns	75 / 90 *U* = 3.00 (.002), *r* = −.73	72 / 90 *U* = 4.00 (.031), *r* = −.52
Emotional Functioning	ns	ns	ns	ns	72 / 95 *U* = 12.50 (.036), *r* = −.51	70 / 95 *U* = 4.50 (.035), *r* = −.51
Social Functioning	ns	85 / 100 *U* = 15.00 (.038), *r* = −.50	ns	ns	85 / 100 *U* = 12.00 (.030), *r* = −.53	
School Functioning	80 / 90 *U* = 11.00 (.014), *r* = −.59	ns	ns	ns	72 / 85 *U* = 7.00 (.008), *r* = −.65	60 / 85 *U* = 4.00 (.029), *r* = −.53
*Parent proxy-report* (*n* = 20)			yes = 3, no = 17	yes = 9, no = 11		yes = 4, no = 16
Summary Score | Total Scale Score	ns	ns	63 / 93 *U* = 5.00 (.030), *r* = −.49	76 / 94 *U* = 18.00 (.016), *r* = −.54	ns	62 / 94 *U* = 5.00 (.011), *r* = −.57
Physical Health Summary Score	ns	ns	59 / 97 *U* = 3.00 (.014), *r* = −.55	69 / 100 *U* = 13.50 (.005), *r* = −.63	ns	56 / 98 *U* = 5.00 (.008), *r* = −.59
Psychosocial Health Summary Score	ns	ns	65 / 92 *U* = 6.00 (.038), *r* = −.46	81 / 93 *U* = 25.50 (.027), *r* = −.49	ns	65 / 92 *U* = 5.50 (.012), *r* = −.56
Emotional Functioning	ns	ns	70 / 90 *U* = 5.50 (.031), *r* = −.48	ns	ns	62 / 90 *U* = 2.50 (.005), *r* = −.63
Social Functioning	ns	ns	80 / 100 *U* = 7.50 (.037), *r* = −.47	80 / 100 *U* = 22.00 (.022), *r* = −.51	ns	70 / 100 *U* = 10.50 (.026), *r* = −.50
School Functioning	ns	ns	ns	ns	ns	60 / 90 *U* = 8.00 (.021), *r* = −.52

Mdn: median; *r*: effect size estimate (from Rosenthal, 1991); ns: non-significant; PedsQL (Scores range from 0 to 100; high scores indicate better health-related quality of life).

### Beliefs and expectations about treatment and perceived HRQoL

Patients who believed that the treatment protects them from disease progression (Mdn = 90) scored higher in the emotional functioning domain (PedsQL) relative to those who had neutral expectations (Mdn = 67, *U* = 7.00, *p* = 0.029, *r* = −0.53). Thirty percent (*n* = 6) of the sample reported feeling anxious on the day of treatment at the hospital (ERT infusion). Patients who reported feeling anxious on this day scored lower (Mdn = 80) on the Physical Health Summary (PedsQL) than those who were not anxious on the day of treatment (Mdn = 100, *U* = 7.00, *p* = 0.029, *r* = −0.45). Scores on social functioning (PedsQL) also showed differed between participants who reported feeling anxious on the day of ERT infusion at the hospital (Mdn = 67) and those who did not feel anxious (Mdn = 98, *U* = 20.00, *p* = 0.047, *r* = −0.44).

Forty-five percent (*n* = 9) of the participants reported the need for more information about the treatment; however, no statistically significant associations were found between the reported level of information about the treatment and HRQoL scores. Eighty percent (*n* = 16) of the sample reported having total confidence in their doctors, although no statistically significant association was found between the level of confidence in the doctors and HRQoL scores.

## Discussion

The reported HRQoL values of the current sample of GD patients and their parents were higher than expected (mean values exceeding 80 on a 0–100 scale). Moreover, the mean scores (all scales of the PedsQL) of the sample were higher than those reported by González-Gil et al. (2012) in children with heart disease and their parents living in Spain. We hypothesize that these results could be attributable to (a) the efficacy of ERT in symptom control in pediatric patients, (b) the fact that all patients were being followed by specialists in hospitals with good health care infrastructure, and (c) the fact that none of the children assessed had severe disease (instead, they had non-neurological type of GD with no splenectomy). Past studies involving adult patients (e.g. Giraldo, Solano, Pérez-Calvo, Giralt, & Rubio-Félix, 2005; Giraldo et al., 2016; Hayes et al., 1998; Masek et al., 1999) demonstrated the positive roles of symptom control, non-splenectomy and ERT in HRQoL assessments.

Our study showed that older children reported worse school functioning (as measured by PedsQL), and female patients reported worse school functioning than male patients. Similar results have been reported in past studies with other congenital diseases (e.g. Fabry Disease; Bugescu et al., 2016). Reasons for that could be related to older children becoming more aware of the long-term consequences of the GD, through comparison with their peers at school. In addition, girls could be more affect by age-related changes leading to missing more days of school, due to overprotection behavior from parents. However, the design of the current study is not appropriate to answer those questions. Future studies need to be conducted to address that questions properly.

Published studies based on the PedsQL in Spain remain scarce; thus, there are no means of comparison and no studies involving healthy children. Furthermore, to the best of our knowledge, this is the first study to assess HRQoL in the pediatric population with GD using validated measures. Previous studies in populations of children and adolescents have focused on neuropsychological functioning, intelligence and cognitive skills (see the introduction section for references) but not on perceived QoL.

Therefore, this study is unique and may provide baseline information for future studies in the field of HRQoL in the pediatric population with GD.

Additionally, the fact that, in this study, HRQoL was measured with the widely used outcome measure PedQL 4.0 ensures that the information provided is relevant and potentially useful for other studies comparing HRQoL across different health conditions. As expected, the PedsQL was easy to apply and effective in the GD population (no missing answers with the protocol), and the information assessed with the generic instrument showed appropriate reliability (i.e. the coefficients for internal consistency exceeded 0.70 for the majority of the subscales in both versions [child self-report and parent proxy-report]). The emotional, social and school functioning subscales in the child self-report showed internal consistency coefficients that were lower than expected (0.58, 0.57 and 0.54, respectively), and the items within these scales showed lower variability for children’s answers, which led to a range restriction that affected the reliability coefficients.

The present study showed that children and parents agreed in their subjective assessments of HRQoL, and the scores obtained by different forms of evaluation did not differ statistically. These results are consistent with those of other studies that used the same instrument (e.g. González-Gil et al., 2012). However, the literature argues that parents and children base their judgments of pediatric HRQoL on different information; therefore, a comprehensive evaluation must consider both perspectives (Eiser & Varni, 2013).

In fact, a detailed examination of our results reveals some discrepancies in the judgments of pediatric HRQoL in the present sample. For example, in children, the presence of symptoms such as bone, joint or abdominal pain had a great impact on reported HRQoL (decreased social and school functioning), whereas in the parents proxy-reports, the presence of such symptoms was not associated with lower levels of quality of life. Similarly, the feeling of being ill and having limitations imposed on involvement in various activities were also perceived by children as substantially negatively impacting quality of life, whereas the parent proxy-reports do not show these effects. In contrast, the parent proxy-reports were affected by the presence of frequent or abnormal bleeding and fatigue in the child, but these conditions were not relevant in children’s reports. Only one condition equally affected the reports of both children and parents: feeling sad. The presence of sadness in the patients had significant negative effects on perceived quality of life.

A literature review (Eiser & Varni, 2013) addressed these differences in perspectives and stated that ‘parents” proxy-reports of their children’s health may be influenced by their own well-being, their involvement in treatment, and their responsibility for the child’s daily care. Parents take into account times when the child is very ill. In contrast, children’s self-reports may be better representations of the immediate effects of disease and treatment on the child’s moment to moment HRQoL and disease-specific symptoms’ (p. 1303).

Moreover, patients’ beliefs about treatment also revealed sources of resilience. Children who reported that the treatment protects them from getting worse showed better emotional functioning than those who had neutral expectations. Indeed, the literature shows that the ability to anticipate positive outcomes is consistently related to psychological well-being and physical health (Salovey, Rothman, Detweiler, & Steward, 2000). Positive expectations for treatment may also increase a child’s sense of control, leading to better emotional regulation.

Additionally, consistent with previous work (Grabowski et al., 2004), our results showed that experiencing pain, fatigue and/or ‘feeling ill’ (restrictions on physical activities) affected social and school functioning.

Patients who reported feeling anxious on the day of treatment (ERT infusion) at the hospital also had lower scores on the physical health and social functioning domains of the PedsQL. Past studies of other congenital diseases have shown that negative emotional states are related to increased difficulties with treatment and poor treatment satisfaction (Remor, 2011) and poorer quality of life (Bugescu et al., 2016). According to patient opinions, living with GD affects their social lives and relationships with friends (Packman et al., 2010).

Although the degree of perceived information about treatment was not related to HRQoL in our study, a group of patients in this study emphasized the need for more knowledge about the treatment, which is consistent with other studies. For example, Packman et al. (2010) noted that

there is a need for increased of education about GD. That is, while they understand that self-education around pertinent GD issues is important, they would like to have healthcare professionals provide more information to them, via websites, pamphlets, and individual or group information sessions (p. 2008).

In the present study, most of the patients reported feeling confident in their doctors, and our data showed no association between the level of confidence in the doctors and quality of life. Although a good relationship with the health care provider may not be directly related to quality of life, past studies (Packman et al., 2010) have reported that doctor-patient communication skills and empathy are highly appreciated and expected from the professionals working with patients with GD.

A limitations of this study is the small sample size, which is often the case for studies on rare diseases. Recruitment based on convenience sampling and the absence of patients with severe disease within the sample are also limitations that may restrict the generalizability of the findings presented here.

In summary, despite its limitations, this cross-sectional study has several strengths. This is the first study assessing HRQoL in pediatric patients with GD and their parents. The selected participants were from different regions of Spain (i.e. includes data from 13 hospitals across the country), and a widely known and robustly validated tool was used for the assessment of HRQoL in the pediatric population considering both children’s and parents’ reports, including completed information on the perceived burden of the disease with additional questions. Moreover, studying the HRQoL in pediatric patients is important since 70% of the clinical symptoms of GD manifest before the age of 18, and approximately 50% of cases are diagnosed before the age of 5.

Finally, the present study shows that children with mild or moderate GD may present good levels of HRQoL when receiving adequate medical treatment and care in a specialized health care facility and are able to control their more relevant symptoms (i.e. pain, bleeding, fatigue, visceral functioning and orthopedic status). However, the subjective experience of being ill, limitations in daily activities, and feeling sad were found to be associated with low HRQoL, with particular adverse effects on social and school functioning. Children and parents cope differently with the disease, and their particular views (i.e. short-term and present versus the long-term perspective) may affect their perceptions and attributions. All of these multidimensional aspects related to HRQoL must be considered to achieve optimal, integrated health care. Psychological and subjective emotional functioning must be considered and not neglected by health care professionals because these functions may be a source of resilience for children and parents facing GD and may contribute to preserving quality of life.

## Supplementary Material

SUPPLEMENTARY_MATERIAL.docx
