# Supplementary material for: Health-related quality of life in children and adolescents living with Gaucher disease and their parents
Source: Health Psychol Behav Med. 2018 Apr 12;6(1):79–92. doi: 10.1080/21642850.2018.1462705 (PMC8114346; doi:10.1080/21642850.2018.1462705)
Supplement: SUPPLEMENTARY_MATERIAL.docx [file RHPB_A_1462705_SM3860.docx]

SUPPLEMENTARY MATERIAL

**Children’s and parents’ HRQoL perspectives**

No statistically significant differences were found between any of the matched domains of the PedsQL according to the perceptions of children (aged 5 to 18 years) and parents. The children and their parents show concordance in their subjective assessments.

Table. Non-parametric test (Wilcoxon signed-rank test) comparing the PedsQL 4.0 scores of children and their parents (n = 17)

| PedsQL 4.0 domains | | | |  |
| --- | --- | --- | --- | --- |
|  | Mdn |  | Mdn | *z* (*p*) |
| **Child Self-Report (aged 5 to 18 years)** |  | **Parent Proxy-Report** |  |  |
| Summary Score \| Total Scale Score | 91.3 | Summary Score \| Total Scale Score | 91.8 | -.39 (.698) |
| Physical Health Summary Score | 93.7 | Physical Health Summary Score | 95.3 | -.04 (.968) |
| Psychosocial Health Summary Score | 88.3 | Psychosocial Health Summary Score | 91.7 | -.63 (.529) |
| Emotional Functioning | 90 | Emotional Functioning | 87.5 | -.46 (.645) |
| Social Functioning | 90 | Social Functioning | 100 | -.72 (.472) |
| School Functioning | 80 | School Functioning | 85 | -.74 (.460) |

Mdn: Median; PedsQL (Scores range from 0 to 100; high scores indicate better health-related quality of life)
